# Applications of Polydopamine-Modified Scaffolds in the Peripheral Nerve Tissue Engineering

**DOI:** 10.3389/fbioe.2020.590998

**Published:** 2020-10-21

**Authors:** Ji Yan, Ruoyin Wu, Sisi Liao, Miao Jiang, Yun Qian

**Affiliations:** ^1^Department of Orthopedics, Shanghai Jiao Tong University Affiliated Sixth People’s Hospital, Shanghai, China; ^2^Shanghai Jiao Tong University School of Medicine, Shanghai, China; ^3^Youth Science and Technology Innovation Studio, Shanghai Jiao Tong University School of Medicine, Shanghai, China; ^4^School of Pharmacy, Shanghai Jiao Tong University, Shanghai, China

**Keywords:** polydopamine, surface modification, peripheral nerve repair, nerve conduit, tissue engineering

## Abstract

Peripheral nerve injury is a common and complicated traumatic disease in clinical neurosurgery. With the rapid advancement and development of medical technologies, novel tissue engineering provides alternative therapies such as nerve conduit transplantation. It has achieved significant outcomes. The scaffold surface modification is vital to the reconstruction of a pro-healing interface. Polydopamine has high chemical activity, adhesion, hydrophilicity, hygroscopicity, stability, biocompatibility, and other properties. It is often used in the surface modification of biomaterials, especially in the peripheral nerve regeneration. The present review discusses that polydopamine can promote the adhesion, proliferation, and differentiation of neural stem cells and the growth of neuronal processes. Polydopamine is widely used in the surface modification of nerve conduits and has a potential application prospect of repairing peripheral nerve injury. Polydopamine-modified scaffolds are promising in the peripheral nerve tissue engineering.

## Introduction

Peripheral nerve injury can lead to partial or total nerve rupture and result in paralysis, neuropathic pain, and even sensory loss. It severely impairs the patients’ limb functions and reduces their qualities of life. Although autologous transplantation is the current gold standard of clinical treatment, it also has many defects, such as limited sources, mismatched diameters, and sensory dysfunction in the donor region (Samadian et al., 2020). Given these problems, the clinical application of nerve conduits is being constantly explored. To fabricate an ideal nerve conduit, the material selection and other important scaffold properties must be considered, such as pro-adhesive interface, conductivity, degradability, biocompatibility, and mechanical stability. Among them, surface modification is a common and vital tool to improve the microenvironment of the cell/scaffold interface to support an adhesive surface. Immobilized bioactive molecules play a significant role in this process (Chen et al., 2016). Studies have shown that physical and chemical properties of the scaffold interface affect the adhesion, proliferation, and differentiation of cells on the biomaterial surface (Wozniak et al., 2004; Bettinger et al., 2009).

Hsiao et al. (2009) attached the synthetic hybrid DNA strands to the plasma membrane of living cells to allow the modification of the cell surface directly. This technique is more suitable for modifying primary cells because prolonged cell culture is not required. A study revealed that the Arg-Gly-Asp (RGD) peptide functionalized bilayers could support neural stem cells (NSCs) attachment and proliferation. The bilayers were prepared through physical absorption. However, this method was inefficient (Ananthanarayanan et al., 2010; Georgiou et al., 2013). Conducting polymers, such as polypyrrole (PPY) and poly(3,4-ethylenedioxythiophene), have been used to facilitate the growth and differentiation of neural cells, based on their electroactivity and electrical conductivity (Rim et al., 2013). However, the mechanical stability is reduced substantially. It also becomes difficult to calculate the exact density and distribution (Green et al., 2009; Gelmi et al., 2010). Besides, it was demonstrated that surface modification was necessary for Schwann cell’s response and polycaprolactone (PCL) could improve biocompatibility (Luca et al., 2014).

Dopamine is a dopa derivative containing catechin groups. Catechol and amino groups in its molecular structure have high chemical activity and play an important role in the adhesion process through covalent bonds and non-covalent forces. Polydopamine (PDA), formed by oxidative self-polymerization of dopamine in weak basic Buer solution, has no special requirements on the properties of materials and can stick to almost any object surface even Teflon (Miller et al., 2017). In 2007, Lee et al. (2007) first discovered a simplified method to apply PDA for material surface modification based on the mussel adhesion protein inspiration. Since then, PDA has been widely used in surface modification of various inorganic or organic materials due to its unique properties, especially in the field of biomedicine. It mainly includes adhesion of organic templates in the biomineralization and preparation of nanocapsules for drug delivery. The biocompatibility and hydrophilicity of the materials were improved as well as the immunogenicity was reduced (Ryu et al., 2010; Cui et al., 2012). In the study of Bhang et al. (2013), it was verified that when the pheochromocytoma (PC) 12 cells were cultured on the PDA-coated surfaces, the expression of marker proteins would be enhanced in the neuronal differentiation, compared to that on the gelatin-modified surfaces. The length of the neurites extended more effectively. Compared with traditional surface modification strategies, PDA coatings have significant advantages. PDA deposition is non-specific, whereas grafting depends on specific sites on the membrane surface. The process of PDA modification is under simple and mild conditions so that it can avoid potential damage in the process of irradiation. The PDA coatings keep scaffolds moist in the plasma treatment that affects the surface permeability of the materials (Miller et al., 2017).

**FIGURE 1:**
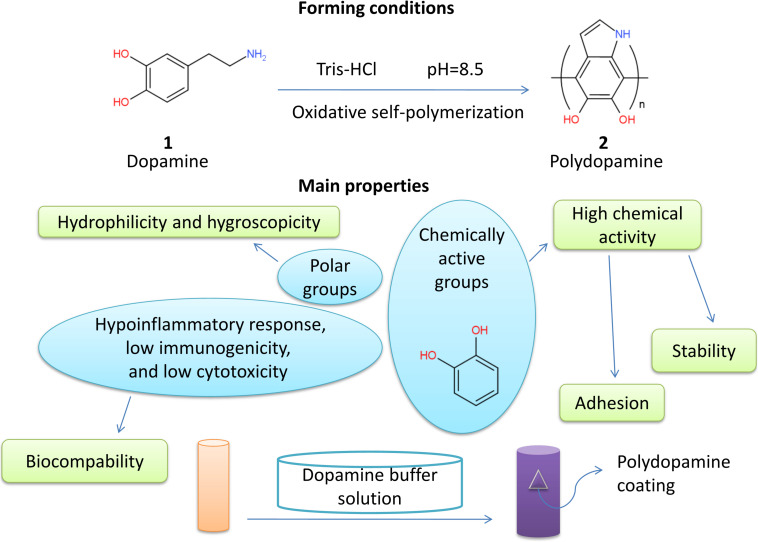
Primary properties of polydopamine.

In recent years, the application of PDA in the nerve tissue engineering has become a research hotspot. In this review, the physicochemical properties, biocompatibility, and effects on neuronal activity of PDA as well as its applications in the peripheral nerve tissue engineering are reviewed (Figure 1).

## Physicochemical Properties of PDA

### Reactions of PDA

Properties of PDA such as adhesion are based on its chemical activity. Chemically active catechol can form hydrogen bonds with hydroxyl and chelate metal ions (Lee et al., 2007). Covalent bonds are commonly formed between catechol and many groups including sulfhydryl and amidogen (Lee et al., 2006).

Polydopamine acts as either oxidant or reductant in a reaction because of its oxidizing quinonyl and the reductive catechol (Miller et al., 2017). Chelation reactions take place when PDA meets metal ions with every valence (Fe^3+^, Zn^2+^, Mn^2+^, Cu^2+^) (Ye et al., 2017). Noble metal ions can be reduced by PDA because the electrons that are released during the oxidation of catechol stimulate the reduction of positive ions (Ball et al., 2011).

When it comes to proteins representative of biomolecules, nucleophilic ones react with carbon on the benzene ring (Michael addition), and the primary amino groups in proteins react with PDA in the quinone form (Schiff base reactions) (Lee et al., 2009). Thiol and amino groups also react with PDA. In alkaline conditions, phenolic groups in PDA are first oxidized to homologous quinone and then react with amino groups by Schiff base reaction or Michael addition. While thiol groups often react with PDA by Michael addition, amino and imino groups in PDA make it more likely to crosslink some organic molecules on PDA (Hu and Mi, 2013; Wang et al., 2013; Liu et al., 2014). Moreover, since PDA hardly bonds to chemical reagents except for water and solutions with metal ions, it shows the advantage when it comes to deposition (Miller et al., 2017).

### Adhesion of PDA

Polydopamine deposits on organic or inorganic materials, regardless of whether the surface is hydrophilic or hydrophobic, and surface properties of composite materials are dominated (Miller et al., 2017). Although adhesion mechanisms are not clear, it is closely related to chemical compositions. There are mainly two kinds of adhesion processes: covalent bindings and non-covalent bindings. Covalent bindings involve Michael addition and Schiff base reaction, and non-covalent bindings include metal coordination, hydrogen bonds, π - π stack, and Van der Waals’ force (Yu et al., 1999; Dalsin et al., 2005; Anderson et al., 2010; Li et al., 2010; Ye et al., 2011; Pop-Georgievski et al., 2012; Liu et al., 2014). The more the non-covalent bonds are, the stronger the adhesion is (Miller et al., 2017). In addition, a high concentration of SO_4_^2–^, NO_3_^–^, and Cl^–^ interrupted deposition. It indicates that adhesion of PDA is selective (Zhang et al., 2018).

After PDA is attached to materials, it performs as a secondary platform for functional molecules (drugs or growth factors, silver nanoparticles, and proteins) binding to the surface (Lee et al., 2009; Ball et al., 2011; Liang et al., 2019). In addition, thicker layers of PDA can bind more drugs with much longer release time (several hundred days), which endows the drugs with sustained release (Yang et al., 2018).

### Hydrophilicity and Hygroscopicity of PDA

Many phenolic and amino groups increase the hydrophilicity of PDA, so that PDA-adhered materials are more hydrophilic, and hydrophobic materials covered by PDA have even much better hydrophilicity (Miller et al., 2017). Because of the existence of strong hydrophilic groups of PDA, such as amino, imino, and catechol groups, the ability of materials to absorb water was improved (Chen et al., 2019).

### Stability of PDA

Stability of PDA depends on synthetic methods. For instance, PDA layers synthesized by air oxidation are heterogeneous and insufficient. They are unstable in polar organic solvents, and acidic and alkaline water solutions. However, PDA layers synthesized by CuSO_4_/H_2_O_2_ in alkaline conditions have better homogeneity and pH stability. They also show strong polarity in organic solvents (Zhang et al., 2016). PDA coatings in water solutions often possess excellent mechanical strength and structural stability (Shen et al., 2015; Fang et al., 2019).

## Biocompatibility of PDA

Polydopamine displays high cellular and tissue biocompatibility characteristics (Han et al., 2017). PDA coatings can reduce inflammation, immune reaction, and cytotoxicity (Hong et al., 2011). Oxidative polymerization of dopamine could promote cell adhesion and proliferation on substrates [polylactic acid, poly(lactic-co-glycolic acid) (PLGA), polyurethane(PU), PCL, hydrophobic materials (poly-ethylene, poly-tetrafluoroethylene, and poly-dimethyl-siloxane)] (Ku et al., 2010; Tsai et al., 2011; Rim et al., 2012). Hydrogel directly applied to skin can reduce inflammation if it contains PDA in synthesis (Han et al., 2017). For example, polystyrene/silver/PDA nanoparticles show no cytotoxicity at low concentration and slight cytotoxicity at high concentration, which guarantees a higher cell survival rate (Cong et al., 2014). H_2_O_2_ can degrade PDA nanoparticles effectively, which has also been proved *in vivo* (Liu et al., 2014). Biodegradability strengthens PDA safety.

## PDA and the Activity of Neuron and Neuron-Likecells

### NSC Adhesion Enhanced by PDA

In recent years, PDA has been widely used in the modification of biomaterials due to its desirable cell adhesion. Neural stem cells have the ability of division and self-renewal. Under appropriate conditions, neural stem cells can differentiate into different types of cells, including neurons, to repair and replace damaged nerve cells. Many experiments have proved that NSCs can effectively adhere to the surface of the composites modified by PDA, indicating it is beneficial to repair injured nerves. However, the molecular mechanism of PDA promoting NSC adhesion is still unclear. The addition of PDA can enhance the hydrophilicity (Cheng et al., 2016). It was reported that enhanced hydrophilicity can promote cell adhesion and other cell behaviors (Lin and Fu, 2016). PDA contains many hydrophilic functional groups, such as hydrophilic amino group and hydroxyl group, which can provide hydrophilic group for hydrophobic surface, thus improving the hydrophilicity of the nerve conduits. In addition, vincristine, a kind of cytoskeleton protein, is closely related to local adhesion and regulates cell proliferation. The expression of vincristine in NSCs cultured with PDA was enhanced significantly, and it showed that NSCs were much more proliferative than the non-PDA treatment group (Yang et al., 2020).

### NSC Proliferation and Differentiation Improved by PDA

Postsynaptic density protein-95(PSD-95) protein exists in the postsynaptic membrane. Its content can reflect the development and maturity of synapses. Beta III tubulin is a specific microtubulin of neurons. Glial fibrillary acidic protein (GFAP) is a specific intermediate filament protein in astrocytes. Microtubule-associated protein 2(MAP2) only exists in the skeleton of mature nerve cells. The gradual increase in PSD-95, MAP2, and beta III tubulin and decrease in GFAP can be regarded as the process of neuronal maturation and neuronal development (Hendrickson et al., 2011). Some studies have shown that the PDA modification can increase the hydrophilicity of the scaffold and then enhance the cell adhesion, leading to the increase in cell proliferation (Lin and Fu, 2016).

### Neurite Outgrowth Induced by PDA

The PDA-modified PLGA nanofiber membrane can effectively adsorb IGF-1 without damaging the growth factor activity and has an obvious slow-release effect on IGF-1. It is beneficial to maintain the long-term and stable accumulation of IGF-1 at the nerve injury site. Thus, it can maximize the function of IGF-1 (Qi et al., 2019). IGF-1stimulates the growth of neurite, promotes the proliferation and differentiation of nerve cells, inhibits the apoptosis of nerve cells, and promotes the repair of injured nerve tissues (Table 1).

**TABLE 1:** Functions and properties of different polydopamine-modified biomaterials.

**Cell type**	**Adhesion**	**Viability**	**Proliferation**	**Differentiation**	**Substrate**	**Surface modification**	**Author/year**
Hippocampal neurons	(+)	(+)	/	/	Electrode, insulators	PDA/PLL	Kang et al., 2011
HSCs	(+)	/	(+)	(+)	PU/PDA/ECM	/	Chen et al., 2019
RSCs	(+)	(+)	(+)	/	SG-PCL, MG-PCL	PDA/RGD	Qian et al., 2018a
Mouse C2C12 myoblasts, PC12 neuronal cells	/	(+)	(+)	(+)	Electrodes	PDA/PPY	Kim et al., 2018
PC12 cells	(+)	(+)	(+)	(+)	/	PDA	Bhang et al., 2013
HNSCs	/	/	(+)	(+)	PS, PLGA	NGF, GDNF, YIGSR, RGD-PDA	Yang et al., 2012
RSCs	(+)	/	(+)	(+)	PCL	dECM/PDA	Chen C. et al., 2018
Primary postnatal mouse cerebellar neurons	(+)	(+)	/	(+)	PNF	PDA	Sieste et al., 2018
RNSCs	(+)	(+)	(+)	(+)	PDA-PLGA	NGF	Pan et al., 2018
RSCs	(+)	(+)	/	/	PLLA	PDA/CGO/PPY	Li et al., 2020
Rat PC12 cells, primary rat DRG neurons	/	(+)	/	/	Fe_3_O_4_⋅Rhodamine 6G	PDA	Wang et al., 2020
Rat PC12 cells	(+)	(+)	(+)	(+)	PLGA/CNT	PDA-lam	Nazeri et al., 2020
Rat PC12 cells	(+)	/	(+)	/	CC	PDA	Chen X. et al., 2018
Rat BMSCs and SCs	(+)	(+)	(+)	(+)	PCL/gold	PDA	Qian et al., 2018b

*“(+)”, presentation of characteristics; PDA, polydopamine; PLL, poly-L-lysine; HSCs, human Schwann cells; PU, polyurethane; ECM, extracellular matrix; RSCs, rat Schwann cells; SG, single-layered graphene; MG, multi-layered graphene; PCL, polycaprolactone; RGD, arginyl glycylaspartic acid; PC12, pheochromocytoma 12; PPY, polypyrrole; HNSCs, human neural stem cells; PS, polystyrene; PLGA, poly(lactic-co-glycolic acid); dECM, decellularized extracellular matrix; PNF, peptide nanofiber; RNSCs, rat neural stem cells; NGF, nerve growth factor; PLLA, poly-l-lactic acid; CGO, carboxylic graphene oxide; DRG, dorsal root ganglia; CNT, carbon nanotube; lam, laminin; CC, carbonized cotton; BMSCs, bone marrow mesenchymal stem cells; SCs, Schwann cells.*

## PDA-Related Bioengineering in the Peripheral Nerve Regeneration

Polydopamine has been widely applied in the field of biomedicine because of its excellent biological characteristics. Several recent studies have focused on the application of PDA in the repair of peripheral nerve injuries in order to develop suitable nerve conduit scaffolds.

Polydopamine has influenced the bioactivities of nerve conduits and the adhesion, growth, and differentiation of neural cells *in vitro*. Novel decellularized extracellular matrix (dECM) and PDA-coated 3D printed PCL-based conduits were created for nerve regeneration. The presence of PDA significantly improved the hydrophilicity and mechanical properties of conduits, as well as cellular behaviors and neuronal differentiation of Schwann cells (Chen C. et al., 2018). Similarly, the PDA coating significantly improved the hydrophilicity and cytocompatibility of fabricated carbon scaffolds. Proliferation and differentiation of nerve cells were significantly accelerated under the electrical stimulation (Chen X. et al., 2018). Nazeri et al. (2020) noted longer neuronal adhesion and growth on PLGA/carbon nanotube scaffolds modified via PDA coating. Meanwhile, PDA coating has been demonstrated to facilitate the efficient immobilization of NGF and adhesion peptides onto substrates. The growth factor or peptide-immobilized substrates enhanced the proliferation and differentiation of human NSCs (Yang et al., 2012). Another study also confirmed enhanced neuronal differentiation of nerve growth factor stimulated PC12 cells on the PDA-coated scaffolds (Bhang et al., 2013). As a mussel-inspired bioactive substance, PDA shows good prospects for the modification of nerve conduits, which may be applied in further preclinical studies and clinical trials.

Not only PDA coating on the nerve conduits plays an important role, but PDA combined with other materials contributes to the cell proliferation and the property improvement of nerve conduits. The manufactured PU/PDA/ECM nerve conduits exhibited significantly enhanced hydrophilicity, biodegradability, cell proliferation, and viability (Chen et al., 2019). The joint effect of PDA and RGD was reported to benefit the adhesion and proliferation of Schwann cells and mediate the process of cell signaling for nerve repair (Qian et al., 2018a). Robust copolymerization of PDA and PPY also showed its capability of enhancing the growth and proliferation of neuronal cells and stimulating neurogenesis (Kim et al., 2018).

Aside from *in vitro* work, PDA has also been explored in some *in vivo* studies. Qian et al. (2018a) showed notable locomotor and sensory function recovery in SD rats due to implantation of graphene-based nerve conduit coated with PDA/RGD. In another study, nerve regenerative capacity of PDA-gold/PCL nanoscaffolds in improving myelin sheath growth and functional recovery was observed on SD rats with sciatic nerve defects (Qian et al., 2018b). More sensitive signals from tibia muscles were demonstrated on the rat sciatic nerve when using PDA/PPY-coated electrodes than bare or PPY-coated electrodes (Kim et al., 2018).

Although there are limitations in *in vivo* researches on PDA-based repair of peripheral nerve injury, existing studies have shown its potential in the long-term clinical effect of peripheral nerve regeneration. More pre-clinical researches are in urgent need to translate PDA-dependent peripheral nerve repair into the clinical work.

## Discussion

Reliable and valid treatment with time sensitivity is of great importance. Autologous nerve transplantation has reduced its clinical outcomes due to its limitations. The immune rejection of allotransplantation also affects the normal life and recovery process of patients. It remains an urgent and unsolved problem on how to prepare nerve grafts with excellent biomimetic performance (Pfister et al., 2011; Tamaki, 2014). The substrate materials used for the preparation of nerve conduits range from macromolecular materials to nanomaterials, from non-degradable materials to degradable materials, such as chitosan, the copolymer of lactic acid-hydroxy acetic acid, and silk fibroin. Nerve conduits prepared by composite materials often include polymer materials for surface modification or strength support to promote cell adhesion and nerve repair (Ekdahl et al., 2011). Luca et al. (2014) carried out surface treatment on PCL film and improved its hydrophilicity by hydrolysis and amino hydrolysis. An *in vitro* cell test showed that the adhesion and proliferation of Schwann cells on the film was improved significantly during a short period of time, indicating the importance of surface treatment for the preparation of nerve conduits. Zhang et al. (2019) cross-linked the natural copolymer silk fibroin protein with the regenerated directional silk fibroin solution to form a high-strength mechanical scaffold. In this way, the neurons and Schwann cells of the dorsal root ganglion could migrate with the uniform positioning of the scaffold. Mecobalamine-loaded silk fibroin scaffold could promote the survival and growth of neurons which indicated its high biocompatibility. Since the surface modification method of PDA films was proposed, this polymer material has been widely used in the surface modification of metals, semiconductors, ceramics, and other materials. It gradually develops from functionalization to diversification, especially in the field of biomedical advantages (Liu et al., 2014). As is shown above, due to its unique properties, PDA can promote the adhesion, proliferation, and differentiation of nerve cells, thus supporting the promotion of peripheral nerve injury repair. Although the experimental evidence for the actual application of PDA in the surface modification of nerve conduit is not sufficient, it is believed that PDA has a broad prospect in the peripheral nerve repair.

## Conclusion

Polydopamine based on mussel inspiration has high hydrophilicity, durable anti-corrosion ability, strong adhesion, and high biocompatibility. It has a broad application prospect in tissue engineering and biomedicine. Catecholamine functional groups can be quickly and effectively coated on the surface of various materials for function improvement through DA oxidation and polymerization reaction triggered by alkali. PDA improves the hydrophilicity and stability of the material surface. PDA-modified biomaterials promote cell adhesion, proliferation, and diffusion. The application of PDA in the nerve tissue engineering is developing rapidly. Although the current research on peripheral nerve repair by PDA is mainly based on *in vitro* cell experiments, abundant existing experimental results indicate that PDA plays an important role in this field. Future experimental and translational work is necessary in order to obtain more findings of PDA on the level of clinical applications.

## Author Contributions

YQ, JY, and SL conceptualized and designed the manuscript. RW, JY, and MJ drafted the manuscript, designed the table, and reviewed the literature. SL designed the figure. YQ revised the manuscript. All authors approved the final version.

## Conflict of Interest

The authors declare that the research was conducted in the absence of any commercial or financial relationships that could be construed as a potential conflict of interest.
